# Characterization of Two Dinoflagellate Cold Shock Domain Proteins

**DOI:** 10.1128/mSphere.00034-15

**Published:** 2016-01-13

**Authors:** Mathieu Beauchemin, Sougata Roy, Sarah Pelletier, Alexandra Averback, Frederic Lanthier, David Morse

**Affiliations:** Institut de Recherche en Biologie Végétale and Département de Sciences Biologiques, Université de Montréal, Montreal, Quebec, Canada; Carnegie Mellon University

**Keywords:** RNA binding domain, DNA binding domain, cold shock domain, dinoflagellates, cold shock protein, transcription

## Abstract

Roughly two-thirds of the proteins annotated as transcription factors in dinoflagellate transcriptomes are cold shock domain-containing proteins (CSPs), an uncommon condition in eukaryotic organisms. However, no functional analysis has ever been reported for a dinoflagellate CSP, and so it is not known if they do in fact act as transcription factors. We describe here some of the properties of two CSPs from the dinoflagellate *Lingulodinium polyedrum*, *Lp*CSP1 and *Lp*CSP2, which contain a glycine-rich C-terminal domain and an N-terminal cold shock domain phylogenetically related to those in bacteria. However, neither of the two *Lp*CSPs act like the bacterial CSP, since they do not functionally complement the *Escherichia coli* quadruple cold shock domain protein mutant BX04, and cold shock does not induce *Lp*CSP1 and *Lp*CSP2 to detectable levels, based on two-dimensional gel electrophoresis. Both CSPs bind to RNA and single-stranded DNA in a nonspecific manner in electrophoretic mobility shift assays, and both proteins also bind double-stranded DNA nonspecifically, albeit more weakly. These CSPs are thus unlikely to act alone as sequence-specific transcription factors.

**IMPORTANCE** Dinoflagellate transcriptomes contain cold shock domain proteins as the major component of the proteins annotated as transcription factors. We show here that the major family of cold shock domain proteins in the dinoflagellate *Lingulodinium* do not bind specific sequences, suggesting that transcriptional control is not a predominant mechanism for regulating gene expression in this group of protists.

## Abstract (precis)

Dinoflagellate transcriptomes contain cold shock domain proteins as the major component of the proteins annotated as transcription factors. We show here that the major family of cold shock domain proteins in the dinoflagellate *Lingulodinium* do not bind specific sequences, suggesting that transcriptional control is not a predominant mechanism for regulating gene expression in this group of protists.

## INTRODUCTION

Cold shock domains (CSD) are an ancient and conserved nucleic acid binding module (1). They are small, roughly 70 amino acids in length, contain 2 amino acid motifs that are shared by RNA recognition motif domains, and have been shown to bind both DNA (2, 3) and RNA (4). They are found in some archaea, in eubacteria, plants, animals and some fungal lineages. However, the roles played by the CSD depend on the organism and on specific domains associated with it. In bacteria, the cold shock response includes a transient and global translation arrest (5), during which time the cells synthesize a small number of cold-inducible proteins and begin reprogramming their translational machinery to accommodate growth at lower temperatures (6). Cold shock proteins (CSPs), which in bacteria consist only of a CSD, are among the few proteins whose synthesis increases after a 15°C cold shock (7). Many of these bacterial CSPs appear to be functionally redundant, as it is necessary to delete four of the nine *Escherichia coli* csp genes before growth at cold temperatures is impaired (8).

The nucleic acid binding properties of CSPs are an integral part of the bacterial cold shock response. For example, *E. coli* CspA stimulates transcription of the DNA gyrase *gyrA* (9), an effect attributable to binding of specific DNA sequences in the *gyrA* promoter (3). In addition, CspA can aid transcription by acting as an antiterminator, an effect suggested to rely on CspA binding to single-stranded regions of the newly synthesized RNA (10). CSP binding to RNA has also been proposed to melt RNA secondary structures that form due to reduced temperature (11). This may improve translation by eliminating inhibitory secondary structures and may also relieve transcriptional stalling due to the formation of stem-loop secondary structures in the nascent RNA. Lastly, CspA can bind its own transcript and protect it from degradation, as the half-life of the message increases roughly 100-fold at 15°C (12). Thus, in *E. coli*, most of the effects produced by the CSP appear to rely on a capacity for binding RNA.

Plant CSPs also contain a CSD, but in addition include a C-terminal glycine-rich domain interspersed with a variable number of CCHC-type zinc finger DNA binding domains (13, 14). The model plant *Arabidopsis thaliana* has four CSD-containing proteins, some of which are upregulated by exposure to cold (13). *A. thaliana* overexpressing selected CSPs showed increased tolerance to freezing (15) and drought (16) through an mRNA chaperone activity (17). Some (but not all) *Arabidopsis* CSPs also complement the cold-sensitive *E. coli* quadruple *csp* mutant (18). However, these CSPs seem to play a broader role as regulators of embryo development, seed germination, and flowering (19, 20). A similar function may be performed by two CSPs from *Oriza sativa*, as the highest expression is found in flowers and seeds (21).

The core CSD in animal CSPs is surrounded by an N-terminal alanine-proline-rich domain and a C-terminal domain with alternating acidic and basic regions. The first example, a protein called YB-1, was identified due to its ability to bind the Y-box (CTGATTGGCCAA) in promoters of major histocompatibility locus genes (2), yet was subsequently revealed to have potent mRNA-stabilizing activity (22). Furthermore, YB-1 has also been shown to regulate translation of specific mRNAs (23), while the frog Y-box protein (FRGY2) was found in ribonucleoproteins particles (RNPs) that sequester maternal mRNAs in oocytes (24).

Recent transcriptomic analyses of dinoflagellates have shown an abundance of CSD proteins (25, 26). These domains are classified as DNA binding by gene ontology (GO) categories, and in dinoflagellate transcriptomes they constitute roughly two-thirds of all the potential transcription factors identified. However, given the many examples of RNA binding by CSPs, it is not clear if the transcription factor classification of dinoflagellate CSPs is accurate. To assess the function of these dinoflagellate proteins, we have expressed and purified two dinoflagellate CSPs, which we named *Lp*CSP1 and *Lp*CSP2, and compared their nucleic acid binding properties with those of the bacterial CSPs to which they are most closely related. Curiously, while able to bind both RNA and DNA in electrophoretic mobility assays (EMSAs), the dinoflagellate proteins do not complement a bacterial CSP mutant and are not induced under cold conditions.

## RESULTS

### *Lp*CSP1 and *Lp*CSP2 are part of a distinct clade in the eukaryotic CSP family.

**FIG 1:**
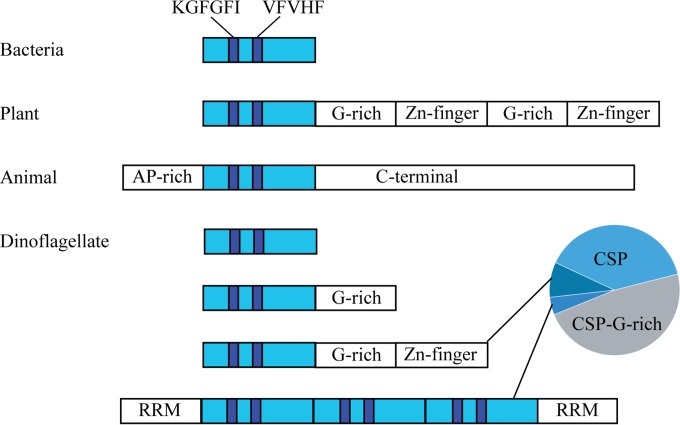
CSPs contain a conserved CSD. The ~70-amino-acid CSD (light blue) is highly conserved in bacteria, plants, animals, and dinoflagellates and contains two RNA recognition motifs (dark blue; KGFGFI and VFVHF). Bacterial CSPs consist only of a CSD, while the other three classes contain C-terminal extensions. The C terminal of plant CSPs typically contains two to seven repeats of a glycine-rich (G-rich) Zn-finger region, a pattern observed in a few dinoflagellate proteins. More typically, when present, the C-terminal extensions of dinoflagellates are G-rich only. An atypical CSD-containing protein found in *Lingulodinium* has three CSD repeats flanked by an RNA recognition module (RRM). Finally, animal CSDs are unique in that they contain an AP-rich N-terminal extension. The pie chart shows the relative abundance of the four different dinoflagellate CSP architectures in the transcriptomes of *Lingulodinium* and *Symbiodinium* species and in the expressed sequence tags of *Alexandrium tamarense* and *Karenia brevis*.

Roughly two-thirds of dinoflagellate proteins classified as transcription factors by gene ontology (GO) are cold shock domain (CSD)-containing proteins (CSPs) (25, 26). The overrepresentation of this class of protein thus suggests they might play an important role in the regulation of gene expression. To begin to address the role of these CSPs, we first determined the types of CSPs expressed by dinoflagellates. We used the CSD in BLAST searches of the transcriptomes of *Lingulodinium* and *Symbiodinium* as well as ESTs from *Alexandrium tamarense* and *Karenia brevis*. We recovered a total of 23 full-length sequences as defined by the presence of a single ORF with in-frame start and stop codons encompassing the CSD, All dinoflagellate CSDs contain the two characteristic RNA binding motifs (KGFGFI and VFVHF) (Fig. 1). However, the CSD itself is found in at least four different domain architectures. The vast majority of the sequences recovered contained a CSD either alone or with a C-terminal G-rich domain (Fig. 1). Smaller numbers of representatives contained a Zn-finger domain following the G-rich domain, and even fewer examples were found of sequences with multiple CSDs and one or more RNA recognition motifs (RRM) (Fig. 1; the pie chart shows distribution of sequence numbers). The domain structure of most dinoflagellate CSPs is thus closest to that found in bacteria or in plants, although there is no sequence similarity between the G-rich domains of plant (27) and dinoflagellate CSPs.

**FIG 2:**
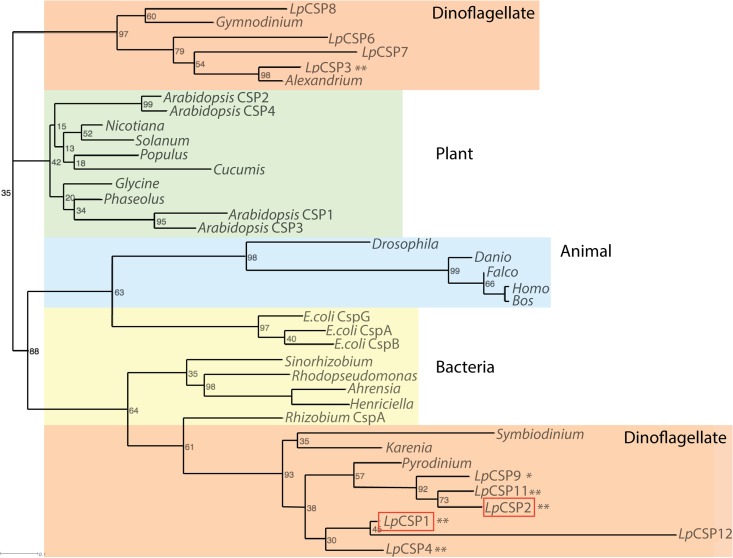
Phylogenetic reconstructions place dinoflagellate CSPs in two distinct clades, one clustered with bacterial and animal sequences and the second in a separate clade unique to dinoflagellates. The *Lingulodinium* CSPs analyzed here are boxed. Sequences marked with a single asterisk contain only a CSD, while those marked with two asterisks contain a CSD and a G-rich domain. The accession numbers for the sequences used are listed in Materials and Methods.

As a complement to the domain structure analysis, a phylogenetic analysis of the CSDs from 10 *Lingulodinium* sequences was also carried out using sequences from a wide array of organisms. In general, support for the different clades was poor, as the CSD is short and the sequence quite conserved. However, there is strong support for grouping the dinoflagellate sequences into two different clades (Fig. 2). One of these two clades also contains both animal and bacterial CSPs, and since the bacterial CSPs are among the best characterized, we elected to begin our analysis with *Lingulodinium* representatives from this clade.

### *Lp*CSP1 and *Lp*CSP2 are not functional complements of *E. coli* CSPs.

**FIG 3:**
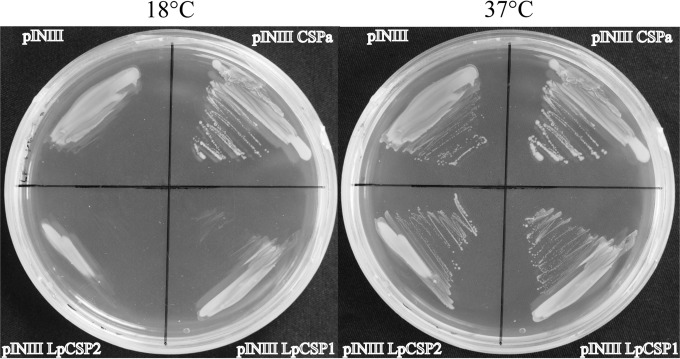
Dinoflagellate CSPs do not complement a quadruple bacterial CSP mutant. Plates were streaked with a quadruple CSP mutant strain BX04 transformed with an empty vector (pINIII), an *E. coli* CspA, *Lp*CSP1, or *Lp*CSP2 and grown first at 18°C for 5 days (left) and then transferred to 37°C overnight (right).

Two dinoflagellate CSPs, which we have termed *Lp*CSP1 and *Lp*CSP2, were cloned and expressed. Both are small proteins (113 and 110 residues, respectively) whose domain structure is similar to that found in the largest class of CSD proteins (an N-terminal CSD followed by a glycine-rich domain). In both, the CSD contains the two expected RNA binding motifs (Fig. 1; see also Fig. S1 in the supplemental material). Bacterial CSPs are required for cell growth at low temperature (28), and a bacterial strain harboring a mutation in four different CSP genes is unable to grow at 17°C (8). However, while overexpression of the bacterial CspA gene into the quadruple mutant allowed for growth at lower temperatures (Fig. 3), neither an empty vector (pINIII) nor the *Lingulodinium* CSPs in pINIII were able to fulfill this role. These dinoflagellate CSPs thus differ from the bacterial CSPs.

10.1128/mSphere.00034-15.2 Figure S1  Sequence alignment of CSD proteins. Sequences of 18 different CSD proteins from a wide diversity of organisms were aligned using MUSCLE. Conserved residues important for RNA binding are highlighted. Accession numbers are listed in Materials and Methods. Download Figure S1, DOCX file, 0.4 MB. Copyright © 2016 Beauchemin et al. 2016 Beauchemin et al. This content is distributed under the terms of the Creative Commons Attribution 4.0 International license.

### *Lp*CSP1 and *Lp*CSP2 expression are not detectable after cold shock.

**FIG 4:**
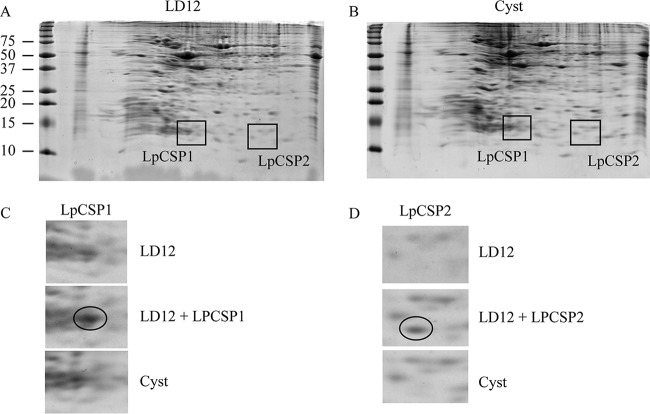
Cold treatment does not induce detectable levels of *Lp*CSP1 and *Lp*CSP2. 2D-PAGE analysis results are shown for *Lingulodinium* total proteins from cells grown at 18°C and collected at LD12 (A) and from cysts harvested after 24 h at 8°C (B). (C and D) The boxed areas in panels A and B correspond to the position of *Lp*CSP1 (C) and *Lp*CSP2 (D) are shown from both gels and from a gel (middle panel) in which 150 ng of recombinant protein was added before isoelectric focusing (circled area). Molecular masses in kilodaltons were estimated using Precision Plus protein standards (Bio-Rad).

Since bacterial CSPs are strongly induced by cold temperature, we also tested if *Lp*CSP1 and *Lp*CSP2 induction could be detected by two-dimensional (2D) PAGE, as seen in *E. coli* (29). The encystment of *Lingulodinium* as a cellular response to cold temperatures (8°C) has previously been reported (30), and we therefore compared the protein profile of cells grown at normal culture room temperatures (Fig. 4A) with those of cysts at 8°C (Fig. 4B). To determine the expected position of *Lp*CSP1 and *Lp*CSP2, 150 µg of *Lingulodinium* protein sample was spiked with 150 ng of purified *Lp*CSP1 or *Lp*CSP2 before electrophoresis (Fig. 4C and D). No detectable induction of *Lingulodinium* CSPs was observed after 24 h at 8°C.

### *Lp*CSPs bind nucleic acids *in vitro.*

**FIG 5:**
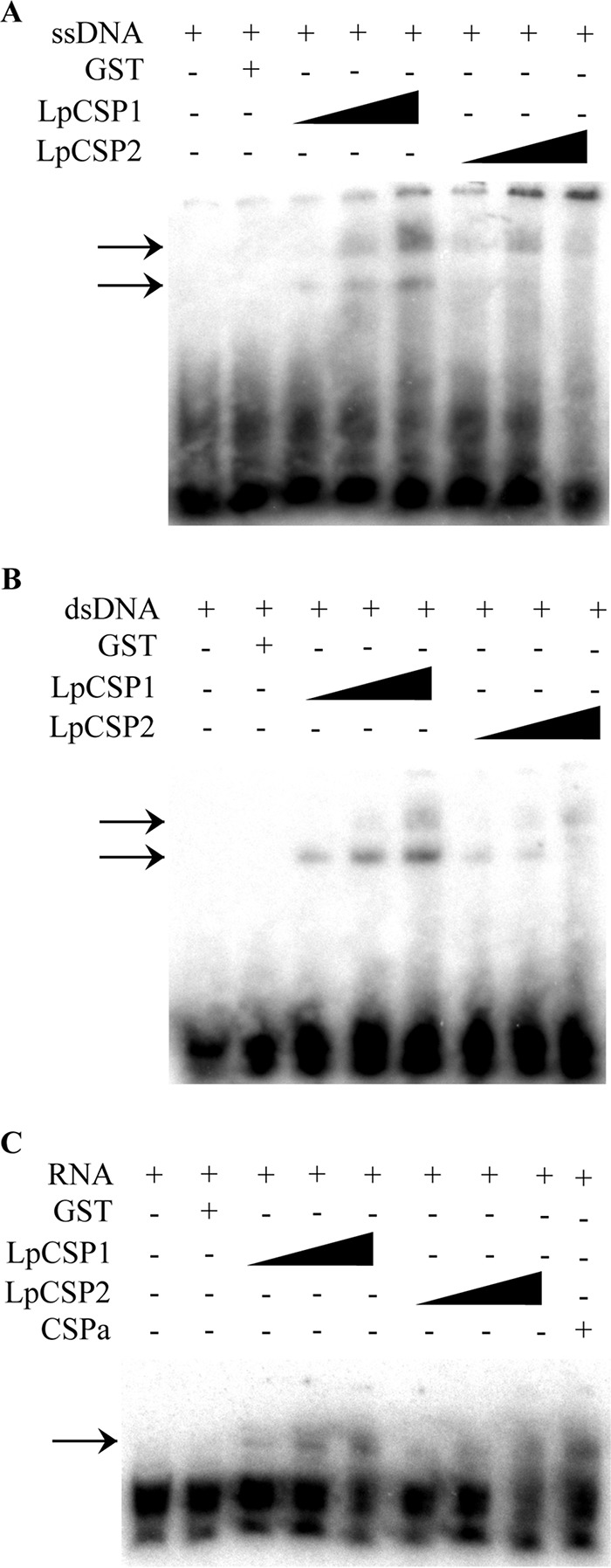
*Lp*CSP1 and *Lp*CSP2 show similar nucleic acid binding activity in EMSA. The EMSAs were carried out using an ssDNA probe (A), a dsDNA probe (B), and an RNA probe (C). The black triangle above the autoradiograms denotes the different concentrations of the CSP recombinant protein used for the assays (100, 300, and 1,000 ng in all the assays; 1,000 ng of CSPa in panel C). Arrows show the bands corresponding to shifted nucleic acids.

Binding of DNA and RNA was evaluated for *Lp*CSP1 and *Lp*CSP2 via EMSA. Both proteins were expressed as glutathione *S*-transferase (GST) fusions and purified, and the GST domain was removed by thrombin digestion prior to use. However, since some residual GST remained in the purified protein fraction (see Fig. S2 in the supplemental material), GST alone was also tested for binding under the same conditions. When single-stranded DNA (ssDNA) was used as a probe, both *Lp*CSP1 and *Lp*CSP2 showed the concentration-dependent slower-migrating bands on EMSA, indicative of nucleic acid binding (Fig. 5A). However, a considerable amount of radiolabeled DNA remained in the loading wells, suggestive of binding by a multimeric CSP to several probe molecules. Both proteins were also able to bind double-stranded DNA (dsDNA), although in this case all bound radiolabel was observed to migrate into the gel (Fig. 5B). Lastly, binding to radiolabeled RNA was also detected for both proteins, with binding similar to that observed for an authentic *E. coli* CSP (Fig. 5C). Both the two *Lingulodinium* proteins thus displayed a broad nucleic acid binding capacity.

10.1128/mSphere.00034-15.3 Figure S2  LpCSP1, LpCSP2, and CspA purification. Recombinant proteins were analyzed on an 18% acrylamide SDS-PAGE gel to assess the purity of CSPs after cleavage of the GST tag with thrombin and binding of the GST to gluathione-Sepharose 4B beads (supernatant lane). Fractions corresponding to a wash and an elution of the beads are also shown to demonstrate cleavage efficiency. Protein sizes in kilodaltons were estimated by comparison to molecular mass markers (Precision Plus Protein Standards; Bio-Rad). Download Figure S2, DOCX file, 0.3 MB. Copyright © 2016 Beauchemin et al. 2016 Beauchemin et al. This content is distributed under the terms of the Creative Commons Attribution 4.0 International license.

To assess the possibility that *Lp*CSP1 might display a capacity for sequence-specific binding, different competitors were added to *Lp*CSP1 in the presence of an ssDNA probe (see Fig. S3A in the supplemental material). These competition experiments indicated that dsDNA competes poorly for binding to the ssDNA and that altering the sequence of the competing ssDNA does not affect its ability to compete. In contrast, ssDNA is an effective competitor of the binding between *Lp*CSP1 and a dsDNA probe (see Fig. S3B). *Lp*CSP1 thus prefers ssDNA substrates.

10.1128/mSphere.00034-15.4 Figure S3  *Lp*CSP1 shows a preference for ssDNA over dsDNA. EMSAs were carried out using an ssDNA probe (A) or a dsDNA probe (B) and 600 ng of *Lp*CSP1. Specific and nonspecific competition were assessed by adding 50× unlabeled probe or 50× cold oligonucleotides (A) or salmon sperm DNA (B). The black triangle above the autoradiograms denotes the addition of unlabeled dsDNA to the radiolabeled ssDNA (A) or of unlabeled ssDNA to the radiolabeled dsDNA probe (B) at amounts corresponding to 1×, 10×, 30×, and 100×. Arrows show the bands that correspond to shifted nucleic acids. Download Figure S3, DOCX file, 0.2 MB. Copyright © 2016 Beauchemin et al. 2016 Beauchemin et al. This content is distributed under the terms of the Creative Commons Attribution 4.0 International license.

## DISCUSSION

In this report, we have assessed the role of two dinoflagellate CSPs. CSPs are potentially important in dinoflagellates, since high-throughput transcriptome studies of *Lingulodinium* (25) and *Symbiodinium* (26) have shown that the majority of the proteins annotated as transcription factors have CSDs. Despite this annotation, however, it is not clear whether any of the dinoflagellate CSPs actually play a role in transcription. To date, with the sole exception of a TATA binding protein (TBP)-like protein, which replaces the TBP usually found in eukaryotic TFIID complexes (31), no transcription factor has been described and characterized experimentally in dinoflagellates.

Two types of CSPs are likely predominant in dinoflagellates, based on the protein domain structure. One form contains only the CSD, while the other contains the CSD and a C-terminal G-rich domain (Fig. 1). Although this analysis is based on the frequency of finding different types of sequence within a transcriptome, it seems likely that the more-frequently found forms would result in a greater amount of protein. The cellular *Lingulodinium* CSP pool may thus be dominated by these two forms, although we cannot rule out the possibility that a protein with a different domain structure could be highly expressed.

Molecular phylogeny of the *L. polyedrum* CSD sequences indicates they are found in two well-supported clades (Fig. 2), perhaps indicative of a functional diversity among dinoflagellate CSPs. We have examined the properties of two members of the group most closely related to bacterial CSPs, *Lp*CSP1 and *Lp*CSP2 (Fig. 2). Despite this relationship with bacterial sequences, however, neither protein appears functionally equivalent to those in bacteria. This was most clearly shown through its inability to complement the growth of the mutant *E. coli* BX04 strain at low temperatures (Fig. 3). The nonspecific mRNA binding activity of bacterial CSPs is the key to their chaperone activity during cold stress (1), so the lack of complementation by *Lp*CSP1 and *Lp*CSP2 is puzzling, given that both dinoflagellate proteins are able to bind an RNA probe *in vitro* (Fig. 5C). However, comparison of two similar *Arabidopsis* CSPs, each containing an N-terminal CSD and a C-terminal G-rich domain, showed that only one was able to complement the BX04 mutant (18). It is thus possible that small differences between sequences are sufficient to disrupt the RNA chaperone activity, which in turn suggests that other dinoflagellate CSD-containing proteins could show RNA chaperone activity and potentially rescue the BX04 strain. However, it is also possible that the lack of complementation in the bacterial assay may reflect the amount of the *Lingulodinium* CSPs actually expressed in the bacteria or an element of sequence specificity that remained undetected in our *in vitro* assays.

We find no evidence that *Lp*CSP1 and *Lp*CSP2 are induced by exposure to cold temperatures. Transcription of both *Arabidopsis* and *E. coli* CSPs can be induced during cold shock (18, 32), but a recent comparison of the transcriptomes of normally growing and cold-shocked *L. polyedrum* cells showed no induction of CSP transcripts by the cold treatment (30). In addition, exposure to cold induces CSP at the protein level in both prokaryotes and eukaryotes, in keeping with their ability to prevent cells from freezing, but a global analysis of the proteome of cold-shocked *L. polyedrum* showed no difference, compared to normally growing cells (30). This agrees with the 2D gel analysis results shown here, where we specifically looked for *Lp*CSP1 and *Lp*CSP2 induction at low temperatures (Fig. 4). Thus, unlike the bacterial CSPs (7, 29), there is no induction of *Lp*CSP1 or *Lp*CSP2 mRNAs, nor are more proteins synthesized, although we cannot rule out a potential posttranslational modification that could modify the position of CSPs spots on a 2D gel. Taken together, a role in cold tolerance seems unlikely for these dinoflagellate CSPs.

Unlike the bacterial CSPs, whose cellular role seems primarily to block formation of extensive RNA secondary structure at cold temperatures (10, 33), CSPs in vertebrates and plants display a number of different roles. The mammalian Y-box binding protein YB1, which shares 40% amino acid identity with the bacterial CSPs (34, 35), has diverse physiological roles apart from the cold stress response (14). YB1 is known to regulate transcription (36, 37) by binding to duplex DNA containing a Y-box (CTGATTGGCT) (2). Similarly, the frog Y-box protein FRGY1 has been shown to stimulate transcription from a promoter containing a Y-box (38). YB-1 has been shown to bind ssDNA with greater affinity than dsDNA (39), and the resulting destabilization of the DNA double helix has been proposed as the transcriptional activation mechanism. However, *Lp*CSP1 and *Lp*CSP2 bind a Y-box dsDNA at very high protein concentrations (300 and 1,000 ng) (Fig. 5B) compared to binding of Y-box DNA sequence by YB1, for which only 30 ng is sufficient (40). The physiological levels of the *Lp*CSP1 and *Lp*CSP2 proteins appear much lower than this, as indicated by our 2D gel profiles of normally growing cells (Fig. 4). This binding also does not appear to be sequence specific (see Fig. S3 in the supplemental material), indicating that specific targeting of the Y-box sequence is doubtful. However, it must be noted that we have not yet tested if other DNA sequences might show preferential binding enabling lower concentrations of *Lp*CSPs to be effective. The stronger binding to ssDNA leaves the door open to a potential role for *Lp*CSPs as destabilizers of the DNA double helix, potentially in cooperation with other, more specific transcription factors.

Plant CSD-containing proteins also have a broader role than a simple response to cold shock. The precise molecular mechanism that enables plant CSPs to regulate processes other than acclimation to abiotic stress, such as seed and flower development, is still unknown. However, the interactions of the *A. thaliana* CSP3 with diverse proteins, such as poly(A) binding proteins, ribosomal proteins, and mRNA-decapping protein, suggest an involvement in multiple RNA processing steps (41). A similar role might be envisioned for *Lp*CSP1 and *Lp*CSP2, as they are able to bind RNA (Fig. 5C). Furthermore, the nonspecific RNA binding by *Lp*CSPs is consistent with a role in mRNA packaging and stability (42), an intriguing prospect in dinoflagellates, where particularly long mRNA half-lives have been documented (43).

Taken together, our results are most consistent with the idea that *Lp*CSP1 and *Lp*CSP2 are not sequence-specific transcription factors, contrary to the impression left by the Gene Ontology assignment. This further reduces the already-scarce number of potential transcription factors in dinoflagellates, a fact that fits well with the limited scale of transcriptional variation seen during the circadian cycle (44), as well as in response to nutrient limitation (45, 46) or abiotic stress (30). We suggest that *Lp*CSPs may still be able to participate in the transcription process, potentially by unwinding the DNA helix due to their capacity to bind ssDNA. However, further studies, such as protein localization and identification of binding partners, will be required to define more precisely the role of these proteins in dinoflagellates. It will also be of interest to examine members of the second dinoflagellate clade, in case these CSPs have substantially different properties.

## MATERIALS AND METHODS

### Cell culture.

Unialgal but not axenic cultures of *Lingulodinium polyedrum* (formerly *Gonyaulax polyedra*; strain CCMP1936) were obtained from the Provasoli-Guillard National Center for Marine Algae and Microbiota (Boothbay Harbor, ME) and grown in modified seawater medium (f/2) (47) at constant temperature (18 ± 1°C). The culture room’s light cycle was 12 h with cool white fluorescent light at an intensity of 50 µmol photons m^−2^ s^−2^, followed by 12 h dark. Under these conditions, the beginning of the light period is termed LD 0 and the beginning of the dark period LD 12. Cultures were typically grown to a cell density of 12,000 to 14,000 cells/ml before cell collection by filtration on Whatman 541 paper supported by a Buchner funnel. Cysts were obtained by placing the cultures at 8°C for 24 hours as described previously (30). All cells were either used immediately or frozen in liquid nitrogen and stored at −80°C until further use.

### Sequence alignment and phylogenetic analyses.

The CSP sequences used for sequence alignment and phylogenetic analyses were obtained from the *Lingulodinium* transcriptome deposited at NCBI. A search of the *Lingolodinium* transcriptome deposited at The Marine Microbial Eukaryote Transcriptome Sequencing Project (48) was also done to retrieve complete sequences when needed. Trees were constructed by using an online tool available at http://www.phylogeny.fr (49). In the workflow, multiple sequences were aligned using MUSCLE and curated using Gblocks. Phylogenetic reconstructions were made using PhyML and visualized using TreeDyn. Sequences used for phylogenetic reconstructions were from the genera (or proteins) *Ahrensia* (WP_018687722), *Alexandrium* (GAIT01073544), *Arabidopsis* CSP1 (AEE86603), *Arabidopsis* CSP2 (NP_195580), *Arabidopsis* CSP3 (NP_565427), *Arabidopsis* CSP4 (Q38896), *Bos* (DAA26237), *Cucumis* (XP_004140332), *Danio* (XP_001340141), *Drosophila* (NP_647983), *E. coli* CspA (AAN82813), *E. coli* CspB (AAB61739), *E. coli* CspG (NP_309172), *Falco* (XP_005244100), *Glycine* (XP_003540832), *Gymnodinium* (GAIL01018775), *Henricella* (WP_018146825), *Homo* (EAW48433), *Karenia* (FK848095), *Lingulodinium* 1 (JO733348), *Lingulodinium* 2 (JO729000 and CAMNT_0033837443), *Lingulodinium* 3 (JO730956 and CAMNT_0033776061), *Lingulodinium* 4 (JO734870 and CAMNT_0033828465), *Lingulodinium* 6 (JO720996 and CAMNT_0033712139), *Lingulodinium* 7 (JO766444 and CAMNT_0033724295), *Lingulodinium* 8 (JO761018 and CAMNT_0033635737), *Lingulodinium* 9 (JO730992 and CAMNT_0033829387), *Lingulodinium* 11 (JO736519), *Lingulodinium* 12 (JO732587), *Nicotiana* (P27484), *Pyrodinium* (GAIO01020278), *Rhizobium* (YP_770349), *Phaseolus* (ESW08176), *Populus* (XP_002313723), *Rhodopseudomonas* (NP_948738), *Sinorhizobium* (AAC64672), *Solanum* (XP_006359670), and *Symbiodinium* 1 (GAFO01002801).

### CSP cloning, expression, and purification.

Primers designed from the *Lingulodinium* transcriptome sequences JO733348 and JO729000 (see Table S1 in the supplemental material) (25) were used to amplify what we termed *Lp*CSP1 and *Lp*CSP2, respectively, from a first-strand cDNA reaction product prepared from total RNA extracted from *L. polyedrum* cells by using Trizol (Invitrogen). The reverse transcription reaction was performed with an Moloney murine leukemia virus reverse transcriptase (Clontech) and the 5′ CDS primer A of the SMARTer RACE (random amplification of cDNA ends) kit (Clontech). The sequences were cloned in the pGEM-T vector (Promega) and verified by sequencing. To allow directional cloning into the multiple-cloning site of the bacterial expression vectors pGEX-4T2 (GE Healthcare) and pINIII, a second PCR was performed on the pGEM-T plasmid containing *Lp*CSP1 or *Lp*CSP2 to add proper restriction sites (see Table S1). *E. coli* CSPa was amplified from a pINIII-CspA plasmid (8) and cloned in pGEX4T2 using similar procedures. The correct frame was verified by sequencing, and the sizes of GST-*Lp*CSP1, GST-*Lp*CSP2, and GST-CspA fusion proteins were verified by SDS-PAGE. The pGEX4T2-*Lp*CSP1, pGEX4T2-*Lp*CSP2, and the pGEX4T2-CspA vector were used to transform chemically competent BL21 host cells (Life Technologies).

10.1128/mSphere.00034-15.1 Table S1  Primers used for amplifying and cloning *Lp*CSP1 and *Lp*CSP2 in the pGEX4T2 and pINIII plasmids (restriction sites used for cloning are underlined) Download Table S1, DOCX file, 0.02 MB. Copyright © 2016 Beauchemin et al. 2016 Beauchemin et al. This content is distributed under the terms of the Creative Commons Attribution 4.0 International license.

A single colony of BL21 *E. coli* containing either pGEX4T2-*Lp*CSP1, pGEX4T2-*Lp*CSP2, or pGEX4T2-CspA was inoculated in 5 ml of Luria-Bertani (LB) broth and cultured overnight at 37°C with vigorous shaking. The overnight cultures were transferred to 250 ml of LB medium supplemented with ampicillin (100 µg/ml) and grown at 37°C with vigorous shaking to an optical density at 600 nm of 0.5. At this point, protein expression was induced for 2 h by addition of isopropyl β-d-1-thiogalactopyranoside (IPTG) to a final concentration of 0.2 mM. After harvesting by centrifugation, the bacterial pellets were resuspended in cold phosphate-buffered saline (PBS) (137 mM NaCl, 2.7 mM KCl, 4.3 mM Na_2_HPO_4_, 1.47 mM KH_2_PO_4_) containing 1 mM phenylmethylsulfonyl fluoride (PMSF), 1 mM dithiothreitol (DTT), 1 mM ethylenediaminetetraacetic acid (EDTA), and 0.25% Triton X-100 and broken with a French pressure cell press (Fisher Scientific). The cell lysates were then centrifuged at 10,000 × *g* for 10 min, and the supernatants were incubated with 100 µl of gluathione-Sepharose 4B beads (Promega) for 45 min at room temperature with end-over-end agitation. Beads were washed 4 times in PBS and resuspended in 300 µl of PBS supplemented with 2 units of thrombin at room temperature for 2 h to cleave the GST tag. Supernatants containing the cleaved CSPs were then electrophoresed by SDS-PAGE on an 18% acrylamide gel to assess purity, and protein concentration was estimated using the Bradford assay (BioRad). Aliquots of purified protein were frozen in liquid nitrogen and stored at −80°C until further use.

### Bacterial complementation assay.

BX04, a quadruple deletion *E. coli* mutant lacking four CSPs, was used to assess the role of *Lp*CSP1 and *Lp*CSP2 in allowing growth at low temperature (18°C) (8). The *E. coli* CspA in pINIII and the empty pINIII vector were used as positive and negative controls, respectively. The plasmids were transformed into chemo-competent BX04 cells. A single transformed BX04 colony with the respective plasmids was inoculated in 5 ml of LB medium and then cultured overnight at 37°C with vigorous shaking. The cultures were streaked on a single LB plate with ampicillin (100 µg/ml) and IPTG (0.2 mM) and grown at 18°C for 120 h. Growth was monitored every 24 h, and after 120 h the same plate was incubated overnight at 37°C.

### 2D-PAGE of proteins from cysts, LD12 cells, and thrombin-cleaved *Lp*CSP1 and *Lp*CSP2.

Total protein was extracted from cysts and LD12 cells by using Trizol (Invitrogen) as described elsewhere (30). A total of 150 µg of LD12 or cyst protein, or 150 µg of LD12 protein spiked with 150 ng of either *Lp*CSP1 or *Lp*CSP2, was loaded on separate 7-cm immobilized pH gradient strips (pH 3 to 10; Bio-Rad) as the first dimension. SDS-PAGE gels containing 15% acrylamide were used for the second dimension and were stained overnight with Coomassie brilliant blue G-250 (50).

### Electrophoretic mobility shift assays.

Double-stranded and single-stranded oligonucleotides were designed as described elsewhere (40) and used after high-performance liquid chromatography (HPLC) purification. The ssDNA was a 32-mer, 5′-TCGATCGGGGCGGGGCGATCGGGGCGGGGCGA-3′, and the dsDNA was prepared by mixing equimolar amounts of the 25-mer 5′-GGTGAGGCTGATTGGCTGGGCAGGA-3′ (the Y-box is shown in italics) and its reverse complement. All oligonucleotides were end-labeled with [γ-^32^P]ATP (PerkinElmer) by using polynucleotide kinase (NEB) and the conditions described by the manufacturer. Labeled probes were purified using the QIAquick nucleotide removal kit (Qiagen). Typically, 1 ng radiolabeled ssDNA or dsDNA probe was incubated with the purified proteins for 30 min in 1× binding buffer (2× binding buffer contained 20 mM Tris-Cl [pH 7.0], 50 mM KCl, 1 mM DTT, 20 mM MgCl_2_, and 10% glycerol) at room temperature. Binding was assessed by electrophoresis on 5% native polyacrylamide gels in 0.5× Tris-borate-EDTA (TBE) buffer at 70 V for 1 h at room temperature, followed by overnight autoradiography on a phosphorimager screen (Amersham) and subsequent imaging with a Typhoon Trio+ (Amersham). For competition assays, the binding buffer was supplemented with a 50× excess of unlabeled ssDNA or dsDNA probe for specific competition and a 50× excess of random single-stranded oligonucleotides or salmon sperm DNA for nonspecific competition.

RNA probes were prepared by *in vitro* transcription using the T7 RiboMAX RNA production system (Promega) from a 120-bp dsDNA template that included the 5′-untranslated region of the peridinin chlorophyll *a* binding protein (PCP; GenBank accession number U93077) preceded by the spliced leader sequence (51) and a T7 promoter. The dsDNA templates were degraded after completion of the reaction by using RQI RNase-free DNase (Promega). The purified RNAs were end-labeled by the same procedure used for ssDNA and dsDNA probes, and unincorporated nucleotides were removed by chromatography on a Bio-Gel P10 column (Bio-Rad). Binding assays were performed as described above.
