# Supplementary figures and images for: Characterization of Two Dinoflagellate Cold Shock Domain Proteins
Source: mSphere. 2016 Jan 13;1(1):e00034-15. doi: 10.1128/mSphere.00034-15 (PMC4863620; doi:10.1128/mSphere.00034-15)

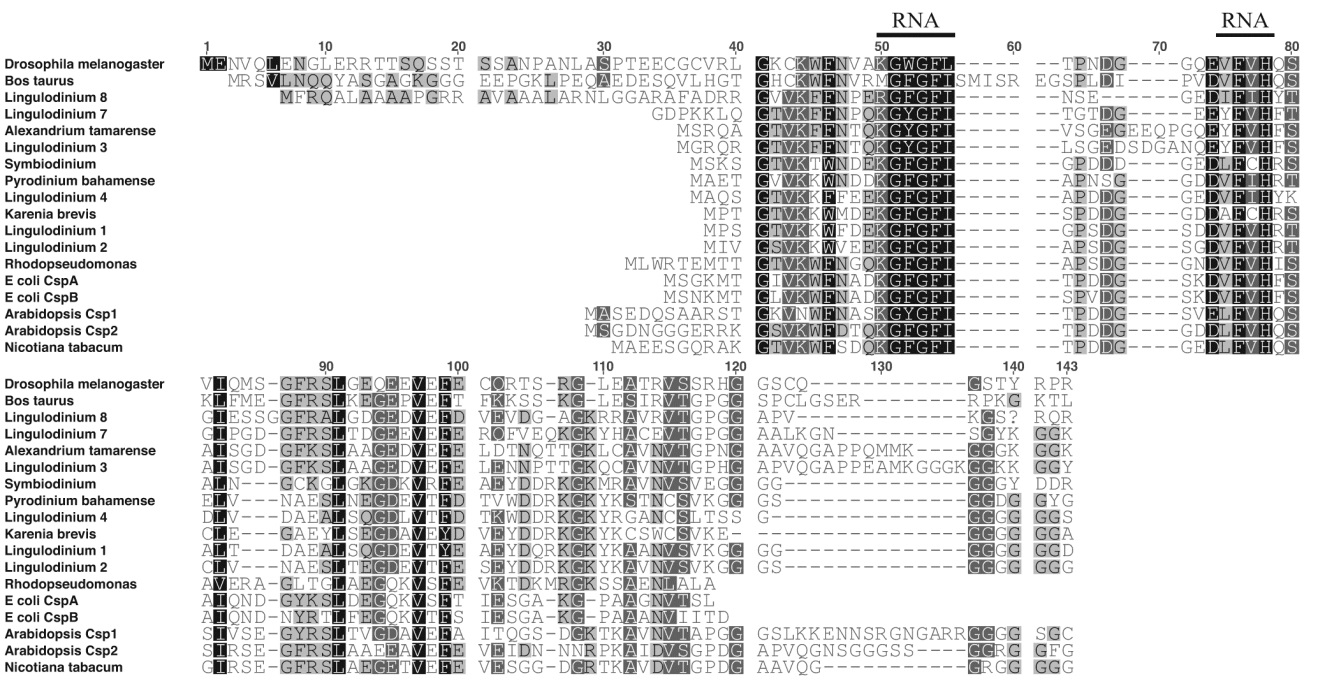


**Supplemental Figure S1** Sequence alignment of CSD proteins

Supplement: Figure S1 [file sph001160057sf2.docx]

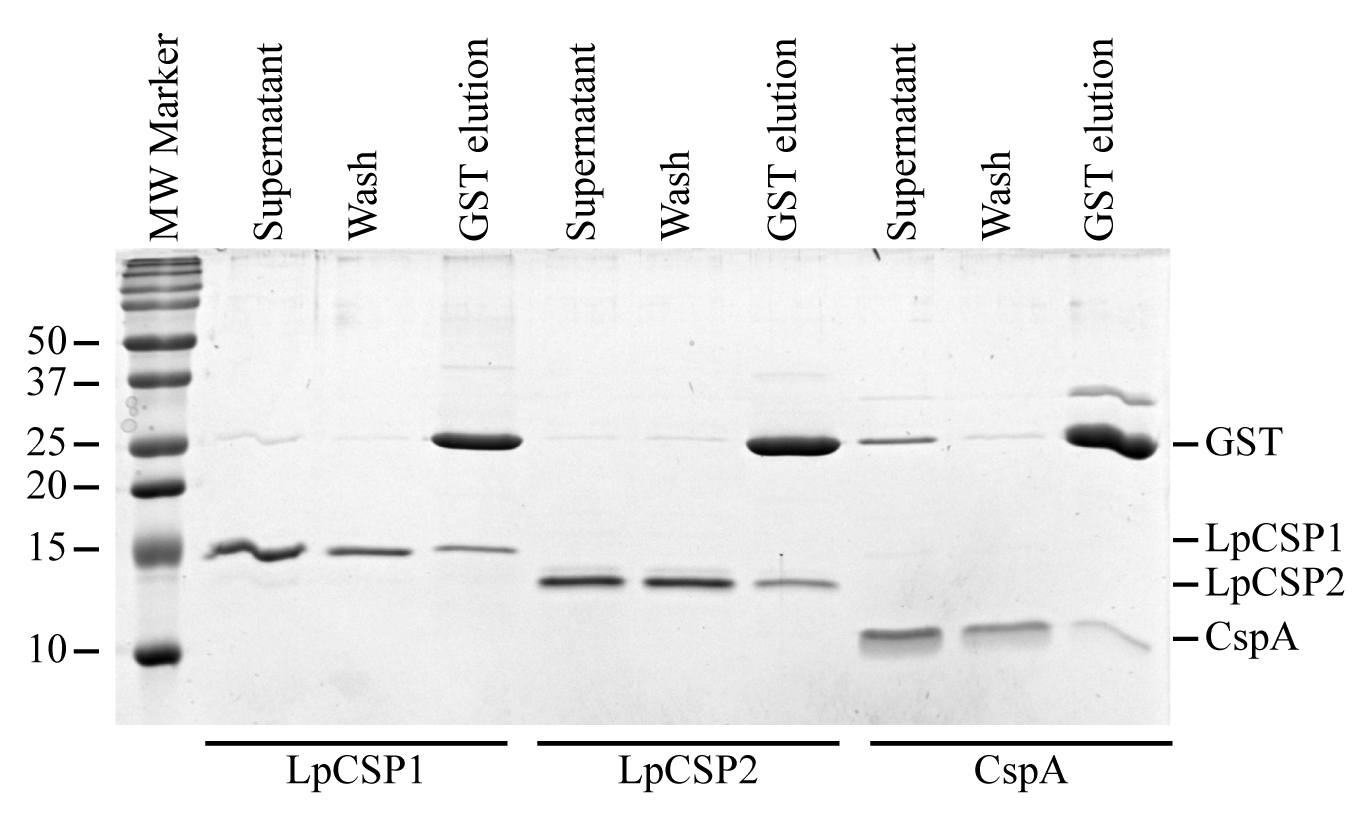


**Supplemental Figure S2** LpCSP1, LpCSP2, and CspA purification

Supplement: Figure S2 [file sph001160057sf3.docx]

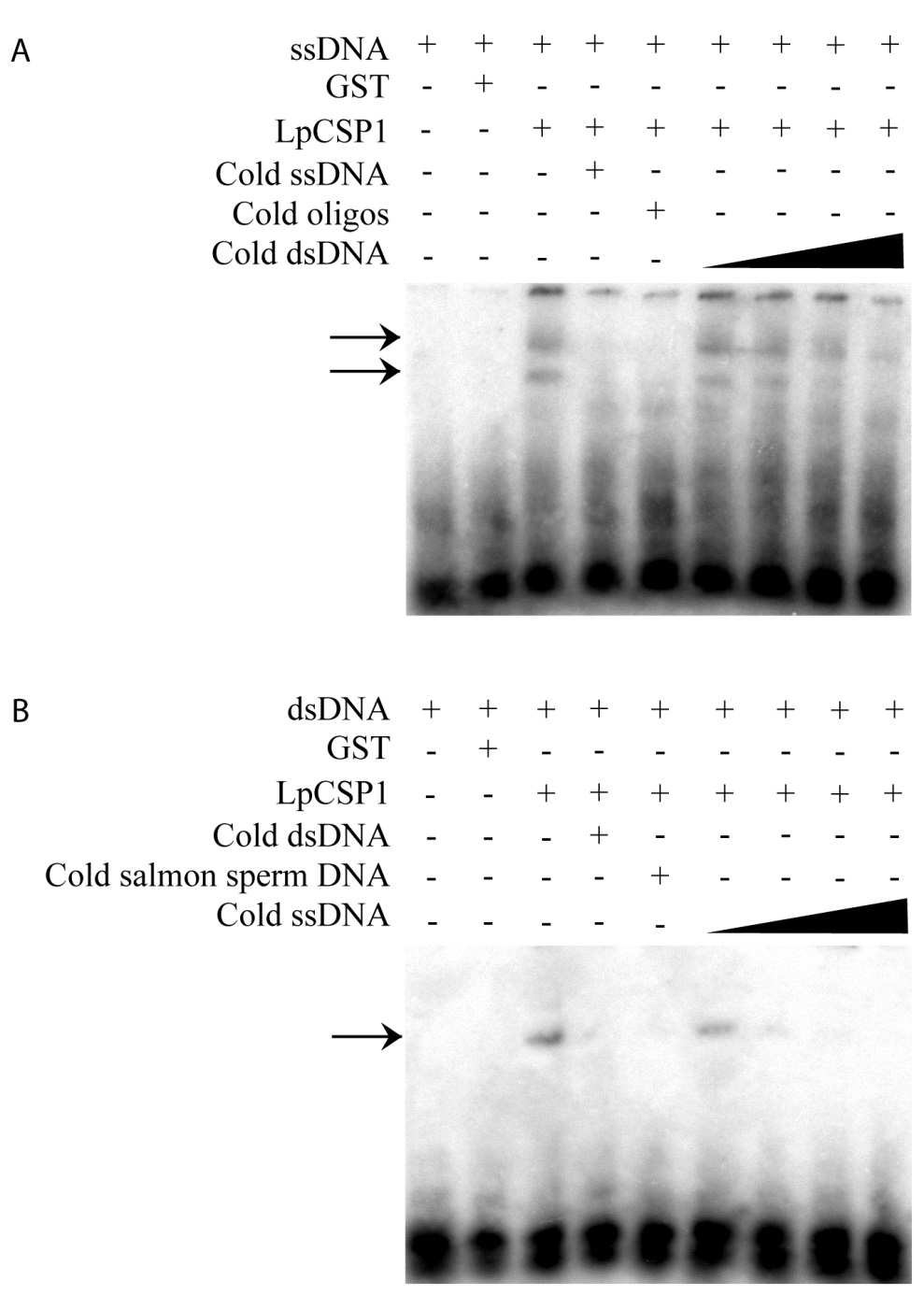


**Supplemental Figure S3** *Lp*CSP1 shows a preference for ssDNA over dsDNA

Supplement: Figure S3 [file sph001160057sf4.docx]
