# Supplementary material for: Characterization of Two Dinoflagellate Cold Shock Domain Proteins
Source: mSphere. 2016 Jan 13;1(1):e00034-15. doi: 10.1128/mSphere.00034-15 (PMC4863620; doi:10.1128/mSphere.00034-15)
Supplement: Table S1 [file sph001160057st1.docx]

Supplemental Table S1

|  | **Accession number** | **Primers**  **name** | **Primers sequence (5’ to 3’)** |
| --- | --- | --- | --- |
| LpCsp1 | JO733348 | CSP1F | gcagcaatgccttccggcactgtgaagaag |
|  |  | CSP1F BamH1 | tgacacGGATCCatgccttccggcactgtgaagaag |
|  |  | CSP1F Nde1 | TGACCATATGccttccggcactgtgaagaag |
|  |  | CSP1R | accctcagctcagaaacctgaggagggtcc |
| LpCsp2 | JO729000 | CSP2F | ATGGCCCAGAGCGGCACGG |
|  |  | CSP2R Xho1 | AACCCTCGAGTCACCAGTAGTCGCCCTTCCC |
|  |  | CSP2F Nde1 | AACCCATATGGCCCAGAGCGGCACGG |
|  |  | CSP2R | TCACCAGTAGTCGCCCTTCCC |
| CspA | NP_418012 | CSPaF | ATGTCCGGTAAAATGACTGGTATC |
|  |  | CSPaR | TTACAGGCTGGTTACGTTACCAG |
|  |  | CSPaR XhoI | AACCCTCGAGTTACAGGCTGGTTACGTTACCAG |
